# Linking person perception and person knowledge in the human brain

**DOI:** 10.1093/scan/nsv148

**Published:** 2016-02-25

**Authors:** Inez M. Greven, Paul E. Downing, Richard Ramsey

**Affiliations:** Wales Institute for Cognitive Neuroscience, School of Psychology, Bangor University, Bangor, Gwynedd, Wales LL57 2AS, UK

**Keywords:** body perception, fMRI, functional connectivity, person knowledge

## Abstract

Neuroscience research has examined separately how we detect human agents on the basis of their face and body (person perception) and how we reason about their thoughts, traits or intentions (person knowledge). Neuroanatomically distinct networks have been associated with person perception and person knowledge, but it remains unknown how multiple features of a person (e.g. thin and kind) are linked to form a holistic identity representation. In this fMRI experiment, we investigated the hypothesis that when encountering another person specialised person perception circuits would be functionally coupled with circuits involved in person knowledge. In a factorial design, we paired bodies or names with trait-based or neutral statements, and independent localiser scans identified body-selective and mentalising networks. When observing a body paired with a trait-implying statement, functional connectivity analyses demonstrated that body-selective patches in bilateral fusiform gyri were functionally coupled with nodes of the mentalising network. We demonstrate that when forming a representation of a person circuits for representing another person’s physical appearance are linked to circuits that are engaged when reasoning about trait-based character. These data support the view that a ‘who’ system for social cognition involves communication between perceptual and inferential mechanisms when forming a representation of another’s identity.

## Introduction

Appreciating the meaning of social interactions depends crucially on understanding others’ identity. For example, one may react differently to an embrace offered from a romantic partner compared with a complete stranger. Attempts to understand the neurocognitive mechanisms that underpin identity processing have focused on two broad research topics: person perception and person knowledge. Person perception research investigates how sensory systems detect conspecifics in the environment on the basis of their face and body (Peelen and Downing, 2007), whereas person knowledge research investigates how inferential mechanisms represent others’ mental states, such as beliefs, desires and attitudes (Frith and Frith, 1999). However, little is currently known about the interaction between social perception and knowledge systems in the human brain. The current fMRI study uses functional connectivity analyses to investigate how distinct neural substrates are linked when perceiving and reasoning about others.

Human neuroimaging studies have provided clear evidence that the processes involved in person perception and person knowledge recruit distinct neural circuits (Adolphs, 2009). Fusiform gyri (FG) and occipitotemporal (OT) cortices respond more to the perception of social (faces and bodies) compared with non-social stimuli (Kanwisher *et al*., 1997; Downing *et al*., 2001; Spiridon *et al.*, 2006), and the majority of evidence suggests that their contribution to understanding identity is restricted to the processing of physical appearance, such as facial features, body shape and posture (Kanwisher, 2010; Downing and Peelen, 2011). A distinct brain circuit comprising medial prefrontal cortex (mPFC), temporoparietal junction (TPJ), precuneus and temporal poles (TPs) has been shown to respond when reasoning about others’ thoughts as well as when making character judgments (Saxe and Kanwisher, 2003; Mitchell, 2009; Schiller *et al*., 2009; Van Overwalle, 2009). The ability to draw inferences about underlying personal characteristics, such as whether someone is hardworking, honest and friendly, also contributes to understanding another’s identity (Ma *et al*., 2012; Macrae and Quadflieg, 2010).

Although it is clear that perceptual and inferential brain circuits contribute to forming an identity representation (Haxby *et al*., 2000; Mitchell *et al*., 2002; Todorov *et al*., 2007), and that trait information can be associated with a person’s physical features, such as their face (Cloutier *et al*., 2011; Mende-Siedlecki *et al*., 2013), a fundamental question in neuroscience is how signals from such segregated neural systems are integrated (Friston *et al*., 2003). Indeed, how integration occurs between the neural representations of others’ physical features and more elaborate cognitive processes remains unclear. For example, functional claims have been made regarding body-selective patches along the ventral visual stream that extend beyond visual analysis of body shape and posture, to include embodiment (Arzy *et al*., 2006), action goals (Marsh *et al*., 2010) and aesthetic perception (Calvo-Merino *et al*., 2010). However, the engagement of body-selective cortical patches in these more elaborate cognitive processes may, in part, index functional coupling within a distributed neural network, rather than local processing alone (Ramsey *et al*., 2011). Our primary focus in the current experiment, therefore, is to test the hypothesis that body patches along the ventral visual stream do not work alone when perceiving and reasoning about others, but interact with extended neural networks.

Prominent models of functional integration in the human brain involve distributed but reciprocally connected neural processing architectures (Mesulam, 1990; Fuster, 1997; Friston and Price, 2001). For example, extended brain networks involving forward and backward connections have been proposed for visual perception of faces (Fairhall and Ishai, 2007), bodies (Ewbank *et al*., 2011), and objects (Bar, 2004; Mechelli *et al*., 2004). Furthermore, when forming identity representations, person perception signals from posterior regions have been proposed to interact with person inference signals from a more anterior circuit (Haxby *et al*., 2000; Ramsey *et al*., 2011; Collins and Olson, 2014).

To date, however, there is little empirical evidence demonstrating interplay between brain systems for person perception and person knowledge. Thus, the current experiment investigates the hypothesis that the representation of identity comprises a distributed but connected set of brain circuits, spanning perceptual and inferential processes. To investigate this hypothesis, we collected functional imaging data while participants were observing two different depictions of an agent (bodies or names) paired with different types of social knowledge (trait-based or neutral). Participants were asked to form an impression of the people they observed. The manipulation of social knowledge replicated prior work that has compared descriptions of behaviour that imply specific traits to those where no trait-based inference can be made (Mitchell, 2009; Cloutier *et al*., 2011; Kuzmanovic *et al*., 2012; Ma *et al*., 2012). In addition, by including two forms of social agent, we are able to investigate the brain circuits that link person knowledge to a specific aspect of a person (physical bodily features), rather than other aspects of a person, which do not engage person perception neural networks, such as a name. By manipulating social agent stimuli and social knowledge information we test a model system of how person perception and person knowledge processes interact in the human brain. We hypothesise that brain circuits involved in person perception and person knowledge will show increased functional connectivity when seeing another person (rather than reading a name) and learning something about his or her trait-based character (rather than trait-neutral information). We expected such tuning to manifest in terms of (i) the magnitude of response observed in body-selective and Theory-of-Mind (ToM) networks, and (ii) the functional connectivity between these networks. This pattern of results would show that when trait inferences are linked to bodies, there is a functional connection between brain regions involved in the visual analysis of body shape and those that are involved in inferring trait inferences and attributing mental states more generally.

## Materials and methods

### Participants

Twenty-three participants were recruited from the Bangor community and received a monetary reimbursement of £10. All participants had normal or correct-to-normal vision and reported no history of neurological damage. They gave informed consent according to the local ethics guidelines. One participant was excluded from data analysis because of a scanner malfunction whilst another was excluded due to difficulties understanding the task. The remaining 21 participants (13 females; mean ± SD age: 24.6 ± 5.7 years) were included in subsequent analyses. For three of these participants, two sessions from the main task had to be removed due to excessive head motion displacement above 3 mm.

### Stimuli and experimental procedure

Participants completed three tasks during scanning: the main experimental task, a body-localiser and a ToM localiser (details of each task are provided below). Each participants’ scanning session started with a run of the body-localiser (4.5 min), followed by two runs of the main task (6 min and 50 s each). This task sequence was then repeated a second time. The body-localiser was interspersed within runs of the main task to introduce a more varied experience for participants and offset boredom. Finally, participants completed two runs of the ToM-localiser (4.5 min each). The ToM-localiser was always presented after the main task, to ensure that participants were not primed towards making trait inferences during the main task. Stimuli were presented using a desktop PC and Matlab software with Psychtoolbox (www.psychtoolbox.org).

#### Main experimental task

**Fig. 1.**
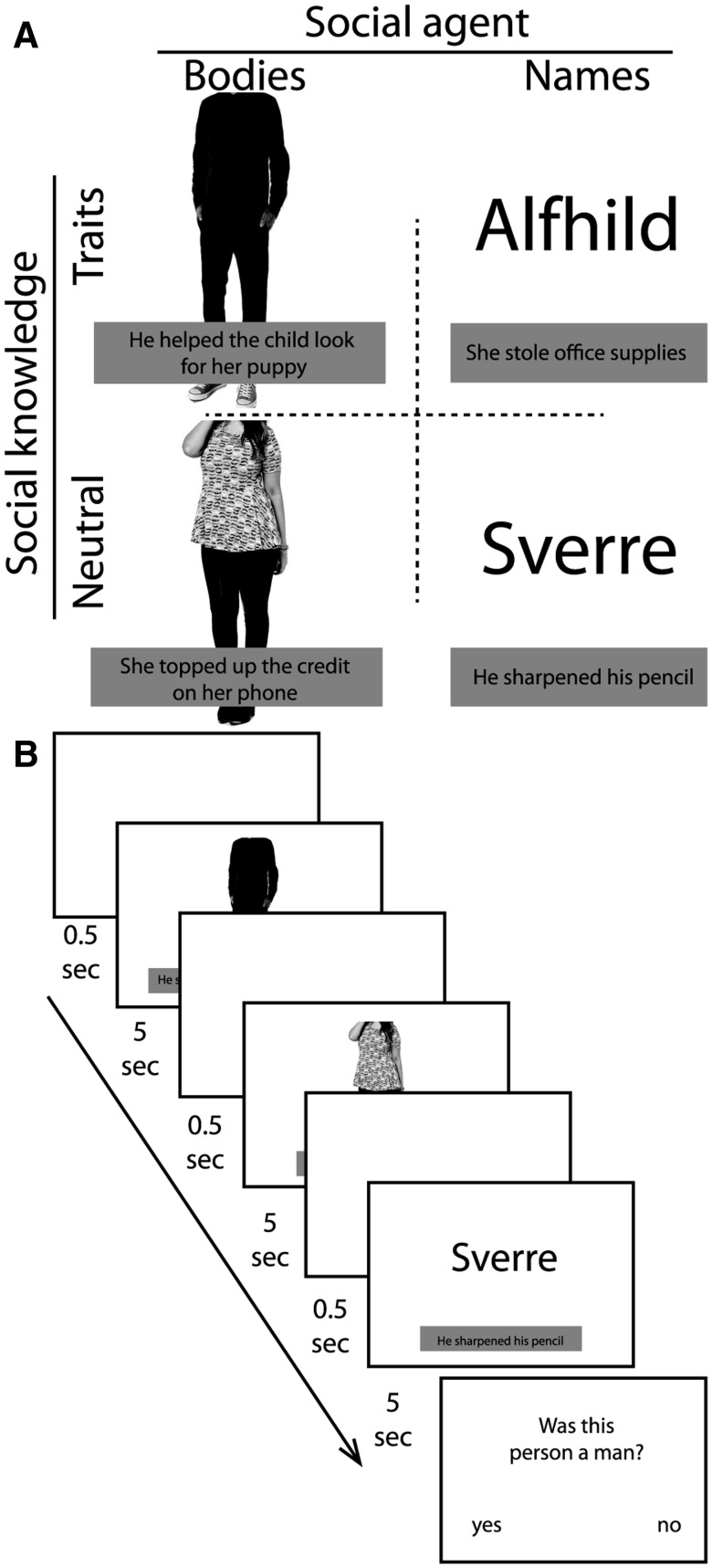
Design and presentation of the stimuli. **(A)** A social agent (body or name) was paired with social knowledge (trait-based or neutral). **(B)** In an event-related design, stimuli were presented for 5 s and separated by a fixation cross for 500 ms. Each block ended with a question about the last trial the participants saw.

The main task comprised an event-related factorial design. In each trial, participants were presented concurrently with a social agent (body or name) and social knowledge (trait-based or neutral) (Figure 1). This resulted in four conditions: bodies paired with traits (BodiesTraits) or neutral statements (BodiesNeutral), and names paired with traits (NamesTraits) or neutral statements (NamesNeutral). For each participant, bodies and names were randomly assigned to the statements. Thus, there was no systematic relationship between particular bodies/names and statements across participants, which removes any coupling between low-level stimulus artefacts and any one condition in our design.

Each trial started with the presentation of a fixation cross for 500 ms, followed by the simultaneous presentation of an agent and a statement for 5000 ms. Participants were instructed to pay attention to both the person (body or name) as well as to the knowledge that they would receive about that person (trait or neutral). There were 256 trials within the entire experiment (64 per condition) that were spread over 4 separate functional runs of equal length. In each functional run, trials were presented in four segments containing a counterbalanced sequence of trials from all four experimental conditions. In order to help effectively model the influence of different events on BOLD signal, one can either introduce jitter between events, or counterbalance the conditions (Josephs and Henson, 1999). We counterbalanced the trial order so that within each segment, each condition was preceded equally often by all conditions (Wager and Nichols, 2003; Aguirre, 2007). To provide a completely balanced trial ‘history’ across conditions, each segment of 16 trials began with a ‘starter trial’, which was not included in the data analysis. Subsequently, four further trials from each condition were presented in a counterbalanced manner. There was a 5-s rest period at the end of each segment.

To make sure participants paid attention to all aspects of the stimuli, at the end of each block they were asked a yes/no-question about the previous trial. Participants were given a response box, which they held with both hands. Within a maximum duration of 5 s, yes/no responses were made by pressing the left and right button, respectively. These questions could be about the agent’s gender (was this person a man/woman?), body (was this person facing forward?), name (was there an a/o in this name?), as well as the person knowledge statements (did this person touch an object? did this person have a positive/negative attitude?). To ensure that participants remained alert to all elements of these stimuli, the content of questions could not be predicted. Accuracy was measured as the percentage of correct answers and compared to chance performance (50%) using 95% confidence intervals (CI.95; Cumming, 2014). Effect size was calculated using Cohen's d by dividing the mean difference from chance performance by the standard deviation pooled across conditions (Cohen, 1992; Lakens, 2013).

Social agent stimuli comprised images of bodies or names. The agent (body: full-colour picture, 300 × 750 pixels; name: black fontcolour, fontsize 60 pt) was presented in the middle of the screen with text (fontsize 30 pt) underneath (250 pixels below the centre of the screen). Pictures of 128 bodies (64 females) were selected that had an emotionally-neutral posture (i.e. crossed-arms or slouching postures were not included) but varied in terms of body shape, skin colour and clothing. Consistent with prior work (Downing *et al*., 2007), in order to target regions selective for images of bodies and not faces, images were cropped so the head was not visible. 128 Scandinavian names (64 females), such as Sverre and Alfhild, were selected to avoid associations with familiar names of people participants may know (Ma *et al*., 2012). Each body and name was only shown once during the entire experiment, to avoid any possible effects of combining the same person with different social knowledge statements over the course of the experiment.

Social knowledge stimuli comprised 128 statements that were adapted from Mitchell *et al*. (2006) to convey either trait-based (positive and negative) or neutral information. An example of a trait-implying statement is ‘He cut in front of the man in line’, implying the person is inconsiderate, whereas a neutral example is ‘She walked through the swivel doors’. Trait and neutral sentences did not differ (as tested with a paired-samples *t*-test) in the mean amount of words [*t*(63) = 0.59, *P* = 0.56], nor in the amount of characters [*t*(63) = 1.69, *P* = 0.09]. Each statement (64 trait, 64 neutral) was presented twice during the experiment (once in female and once in male form; e.g. ‘She walked …’ and ‘He walked …’).

#### Functional localisers

To localise body-selective brain regions we used an established paradigm (Downing *et al*., 2007; http://pages.bangor.ac.uk/∼pss811/page7/page7.html). We presented 12-s blocks of cars and of whole bodies (without heads) that were not used in the main task. A run started with a blank screen for 14 s, followed by two alternations of each condition. This was repeated a second time, and followed by a final rest period of 14 s. Each image was presented for 600 ms, followed by a blank screen for 100 ms. Twice during each block, the same image was presented two times in a row. Participants had to press a button whenever they detected this immediate repetition (1-back task). The image location was slightly jittered (10 pixels around central fixation dot) to prevent participants from performing the 1-back task based on low-level after-effects from the previous image. Each participant completed two runs of this task, each with a complementary order of conditions (if run 1 started with bodies, run 2 would start with cars).

To localise brain regions that respond to mental state reasoning, we used an established ToM-localiser (Dodell-Feder *et al*., 2011; http://saxelab.mit.edu/superloc.php). Participants read 10 short false belief stories, in which the characters have false beliefs about the state of the world. Participants also read 10 false photograph stories, where a photograph, map or sign has out-dated or misleading information. After reading each story, participants had to answer whether the subsequently presented statement was true or false. Each run started with a 12-s rest period, after which the stories and questions were presented for 14 s combined (stories: 10 s; questions: 4 s), and were separated by a 12-s rest period. The order of items and conditions is identical for each subject. In the first run, stimuli 1–5 from each condition were presented. The remaining stimuli were presented during the second run.

For both the body and ToM localiser, a design matrix was fitted for each participant with three regressors, two for each condition (bodies and cars; false beliefs and false photographs) and one for the rest periods. Body-selective regions were revealed by contrasting bodies and cars (Bodies > Cars). The ToM-network was revealed by contrasting false beliefs with false photographs (False Beliefs > False Photographs).

### Data Acquisition

The experiment was conducted on a 3 Tesla scanner (Philips Achieva), equipped with an eight-channel SENSE-head coil. Stimuli were projected on a screen behind the scanner, which participants viewed via a mirror mounted on the head-coil. T2*-weighted functional images were acquired using a gradient-echo echo-planar imaging sequence. An acquisition time of 2000 ms was used (image resolution: 3.03 × 3.03 × 4 mm^3^, TE = 30, flip angle = 90°). After the functional runs were completed, a high-resolution T1-weighted structural image was acquired for each participant (voxel size = 1 × 1 × 1 mm^3^, TE = 3.8 ms, flip angle = 8°, FoV = 288 × 232 × 175 mm^3^). Four dummy scans (4 ×2000 ms) were routinely acquired at the start of each functional run and were excluded from analysis.

### Data pre-processing and analysis

Data were pre-processed and analysed using SPM8 (Wellcome Trust Department of Cognitive Neurology, London, UK: www.fil.ion.ucl.ac.uk/spm/). Functional images were realigned, unwarped, corrected for slice timing, and normalised to the MNI template with a resolution of 3 × 3 × 3 mm and spatially smoothed using an 8-mm smoothing kernel. Head motion was examined for each functional run and a run was not analysed further if displacement across the scan exceeded 3 mm.

#### Univariate model and analysis

Each trial was modelled from the onset of the body/name and statement for a duration of 5 s. A design matrix was fitted for each participant with 6 regressors, one for each condition of the 2 × 2 factorial design (4 in total), one for the discarded starter trials and one for the question at the end of each block. Main effects of social agent (Bodies > Names: BodiesTraits + BodiesNeutral > NamesTraits + NamesNeutral) and social knowledge (Traits > Neutral: BodiesTraits + NamesTraits > BodiesNeutral + NamesNeutral) were evaluated to help demonstrate that our task engaged body-selective and ToM areas, respectively. We also evaluated the interaction of bodies and trait information to test our primary hypothesis [(BodiesTraits > BodiesNeutral) > (NamesTraits > NamesNeutral)].

#### Response magnitude analyses

To test the magnitude-based prediction, we calculated which brain regions showed a greater response for trait inferences (Traits > Neutral) when observing a body compared with reading a name. Two possible forms of interaction are predicted: (i) the effect of social knowledge (Traits > Neutral) will be present for both social agents, but be greater for bodies than names; (ii) the effect of social knowledge (Traits > Neutral) will be present for bodies, but not names. To help distinguish among possible interaction patterns, we exclusively mask our interaction result by (NamesNeutral > NamesTraits). Exclusive masking in this manner makes sure that any interaction result is not produced by an unpredicted preference for neutral over trait-based information when paired with names.

#### Psychophysiological interaction analysis

To test our hypothesis that body-selective cortical regions functionally couple with regions associated with mentalising when one sees a body and also infers a trait from it, we assessed the relationship between these regions using a psychophysiological interaction (PPI) analysis (Friston *et al*., 1997). PPI enables the identification of brain regions whose activity correlates with the activity of a seed region as a function of a task. Here we used a generalised form of PPI, which allows for comparisons across the complete design space, including more than two conditions (McLaren *et al*., 2012). By doing so, it is possible to see whether any voxels across the brain show a correlation with activity in the seed region (the ‘physiological element’) as a function of the four conditions within the main task (the ‘psychological’ element).

**Fig. 2.**
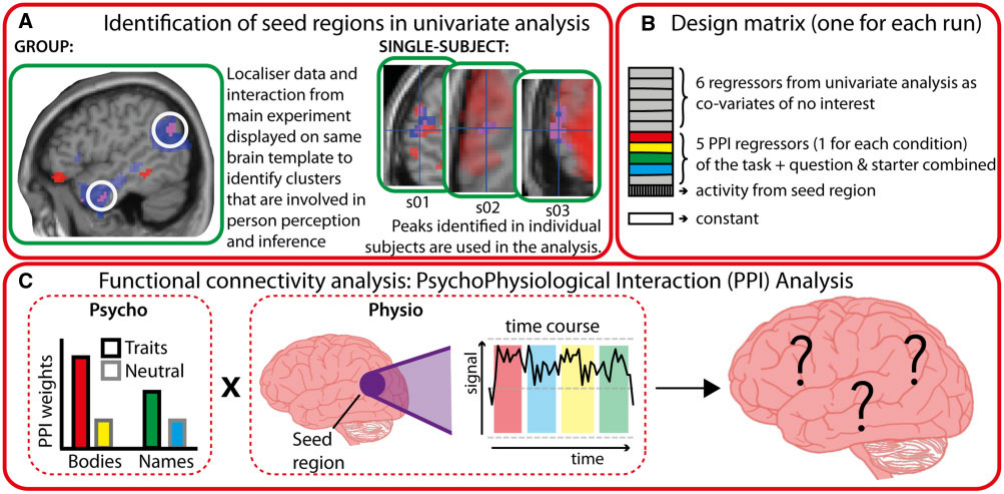
Flow chart illustrating the steps to define seed regions and run PPI analyses. **(A)** Identification of seed regions in the univariate analysis was done at group and single-subject level to allow for inter-individual differences in peak responses. **(B)** An illustration of the design matrix (this was the same for each run), that was created for each participant. **(C)** The ‘psychological’ (task) and ‘physiological’ (time course from seed region) inputs for the PPI analysis.

Our hypothesis was that the same parts of the person perception and person knowledge networks, which show a magnitude-based sensitivity to observing others and inferring traits (revealed in the univariate interaction analysis), would also show functional coupling with each other. As such, seed regions for the PPI analysis were defined based on results from the univariate analysis. Two steps were taken to define seed regions (Figure 2A). First, based on the group-level random-effects univariate analysis, we identified any clusters of overlap between (i) regions in which the type of social agent and social knowledge interacted in the predicted way (in the main experiment) and (ii) either body-selective or ToM-selective regions as identified in the functional localisers. Second, where such clusters of overlap were identified at the group-level, we identified regions of overlap using the same approach in each individual participant. This approach allows us to identify with best possible resolution the key regions where these two phenomena concur. Therefore, regions identified in this manner respond to one of the localisers (Body or ToM), as well as the interaction term in the main task.

In the analyses performed at the single-subject level, we searched for overlap across a range of thresholds, which is common when identifying seed regions in individual’s data (Spunt and Lieberman, 2012; Klapper *et al*., 2014; Paulus *et al*., 2014). For each seed region, therefore, we report how many participants show overlap between the interaction term in the main task (across a range of thresholds) and functional localisers at a fixed threshold [*P* < .005, voxel-extent (k) = 10]. Volumes were generated using a 6-mm sphere, which were positioned on each individual’s seed-region peak.

PPI analyses were run for all seed regions that were identified in each participant. PPI models included the six regressors from the univariate analyses, as well as six PPI regressors, one for each of the four conditions of the factorial design, one for the starter trial and question combined, and one that modelled seed region activity. Although we used clusters emerging from the univariate analysis to define seed regions for the PPI analysis, our PPI analysis is not circular (Kriegeskorte *et al*., 2009). Because all regressors from the univariate analysis are included within the PPI model as covariates of no interest (O’Reilly *et al*., 2012), the PPI analyses are only sensitive to variance in addition to that which is already explained by other regressors in the design (Figure 2B). Thus, the PPI analysis is statistically independent to the univariate analysis. Consequently, if clusters were only co-active as a function of the interaction term from the univariate task regressors, then we would not show any results using the PPI interaction term. Any correlations observed between a seed region and a resulting cluster explains variance above and beyond task-based activity as measured using a standard univariate General Linear Model (GLM).

To create these PPI regressors, the time series in the seed region was specified as the first eigenvariate, and was consequently deconvolved to estimate the underlying neural activity (Gitelman *et al*., 2003). Then, the deconvolved time series was multiplied by the predicted, pre-convolved time series of each of the five conditions 4 main task conditions plus the combined starter trial and question regressor. The resulting PPI for each condition in terms of predicted ‘neural’ activity was then convolved with the canonical haemodynamic response function, and the time series of the seed region was included as a covariate of no interest (McLaren *et al*., 2012; Spunt and Lieberman, 2012; Klapper *et al*., 2014). At the second-level analysis, we examined the same social agent*social knowledge interaction term as described in the univariate analyses [(BodiesTraits > BodiesNeutral) > (NamesTraits > NamesNeutral)].

Names and neutral statements functioned as control conditions within our design. As such, names and neutral statements were included to allow comparisons to bodies and trait-diagnostic statements, and not because we had predictions for how names or neutral information are represented in terms of neural systems (see ‘Discussion’ section for more details). Consequently, the (Names > Bodies), (Neutral > Trait) and inverse interaction [(NamesTraits > NamesNeutral) > (BodiesTraits > BodiesNeutral)] contrasts did not address our main research question. Such contrasts, however, may be useful in future meta-analyses and we therefore report results from these contrasts in Supplementary Table S1.

For all group-level analyses (univariate and connectivity-based), images were thresholded using a voxel-level threshold of *P* < 0.005 and a voxel-extent of 10 voxels (Lieberman and Cunningham, 2009). Based on our hypotheses for functional connections between person perception and person knowledge networks, contrasts from the main task were inclusively masked by the results from the functional localiser contrasts. The results from these analyses are presented in Tables 1 and 2. Results that survive correction for multiple comparisons at the cluster level (Friston *et al*., 1994) using family-wise error (FWE) correction (*P* < .05) are shown in bold font. To localise functional responses we used the anatomy toolbox (Eickhoff *et al*., 2005).

**Table 1.** Results from the univariate analysis.

Region	Number of voxels	*T*	Montreal Neurological Institute coordinates		
			*x*	*y*	*z*
***a) Main effect Social Agent: Bodies > Names***					
**Left occipitotemporal cortex**	**498**	**11.12**	**−45**	**−82**	**−2**
		**6.26**	**−51**	**−70**	**16**
**Right occipitotemporal cortex extending into fusiform gyrus**	**970**	**10.60**	**45**	**−82**	**−2**
		**10.50**	**54**	**−70**	**4**
		**9.92**	**45**	**−76**	**10**
Left hippocampus	50	9.68	−18	−31	−5
Right hippocampus	100	9.01	18	−31	−2
Right inferior temporal gyrus	173	7.23	33	−4	−44
		5.87	30	−4	−35
		5.59	24	−4	−23
Right inferior frontal gyrus	37	6.87	48	35	4
Right cuneus	60	5.64	21	−79	43
		4.74	21	**−**61	58
Right inferior frontal gyrus	16	5.60	24	17	**−**26
Right calcarine gyrus	11	5.41	21	**−**94	22
Left fusiform gyrus	83	5.31	**−**39	**−**49	**−**26
		4.74	**−**36	**−**37	**−**29
		4.55	**−**39	**−**37	**−**20
Striatum	27	5.27	3	11	**−**17
Right inferior frontal gyrus	10	3.95	45	29	13
Left cerebellum	10	3.90	**−**9	**−**55	**−**50
***b) Main effect Social Knowledge: Traits > Neutral***					
**Left temporal pole**	**698**	**11.43**	**−51**	**11**	**−26**
		**10.08**	**−54**	**−1**	**−23**
		**9.23**	**−45**	**26**	**−14**
**Right temporal pole**	**510**	**10.88**	**51**	**11**	**−35**
		**8.68**	**60**	**−4**	**−20**
		**7.63**	**51**	**−13**	**−20**
Left medial prefrontal cortex	442	6.84	**−**3	53	31
		6.01	**−**12	53	43
		4.99	6	65	13
Left inferior frontal gyrus	68	6.40	**−**51	20	7
		5.52	**−**57	23	19
Right cerebellum	120	5.71	24	**−**82	**−**38
Left temporoparietal junction	211	5.06	**−**60	**−**58	19
		3.91	**−**48	**−**58	22
Right medial cerebellum	33	5.00	3	**−**58	**−**50
Right inferior frontal gyrus	46	4.97	48	23	**−**14
		4.49	60	26	7
Precuneus	101	4.74	**−**3	**−**52	28
		3.87	**−**3	**−**61	34
c) Interaction: Social agent * knowledge [(BodiesTraits > BodiesNeutral) > (NamesTraits > NamesNeutral)]					
Left temporal pole	24	4.16	**−**51	**−**1	**−**35
Right superior medial prefrontal cortex	33	4.04	9	50	28
Left superior medial prefrontal cortex	12	3.53	**−**9	32	55
Left temporoparietal junction	15	3.48	**−**48	**−**64	31

The main effect of social agent (Bodies > Names) is masked by the body-localiser (Bodies > Cars); the main effect of social knowledge (Traits > Neutral) is masked by the ToM-localiser (False Beliefs > False Photographs); the social agent by social knowledge interaction [(BodiesTraits > BodiesNeutral) > (NamesTraits > NamesNeutral)] is masked by both the body and ToM localiser.

Note: Regions surviving a voxel-level threshold of *P* < 0.005 and 10 voxels are reported. Areas in bold survive FWE cluster correction for multiple comparisons. Subclusters at least 8 mm from the main peak are listed. The social agent by social knowledge interaction is exclusively masked by the NamesNeutral > NamesTraits contrast to make sure that any interaction result does not include (Neutral > Traits) when paired with names.

**Table 2.** Clusters revealed in the PPI analysis for the social agent by social knowledge interaction [(BodiesTraits > BodiesNeutral) > (NamesTraits > NamesNeutral)], which is masked by both the body and ToM localiser

Region	Number of voxels	*T*	Montreal Neurological Institute coordinates		
			*x*	*y*	*z*
***Seed region: right fusiform gyrus***					
Right temporal pole	11	4.23	51	11	**−**38
Left temporoparietal junction	17	3.79	**−**60	**−**52	19
		2.98	**−**60	**−**46	19
Left temporal pole	14	3.75	**−**45	20	**−**23
		3.28	**−**45	17	**−**32
Left temporal pole	12	3.53	**−**33	5	**−**26
		2.63	**−**42	2	**−**26
***Seed region: left**** temporal pole*						
Left fusiform gyrus	11	4.05	**−**45	**−**49	**−**26

Note: Regions surviving a voxel-level threshold of *P *< 0.005 and 10 voxels are reported. These results are exclusively masked by the NamesNeutral > NamesTraits PPI contrast to make sure that any interaction result does not include (Neutral > Traits) when paired with names.

## Results

### Behavioural data

During the main task, participants’ accuracy was assessed in order to see whether they had been paying attention to the task. Accuracy (percentage correct) in answering the yes/no-questions at the end of each block was above chance-level [*M* = 87.2, CI.95 (82.75, 91.65), Cohen’s *d* = 3.81].

### Neuroimaging data

#### Univariate analyses

##### Main effects

**Fig. 3.**
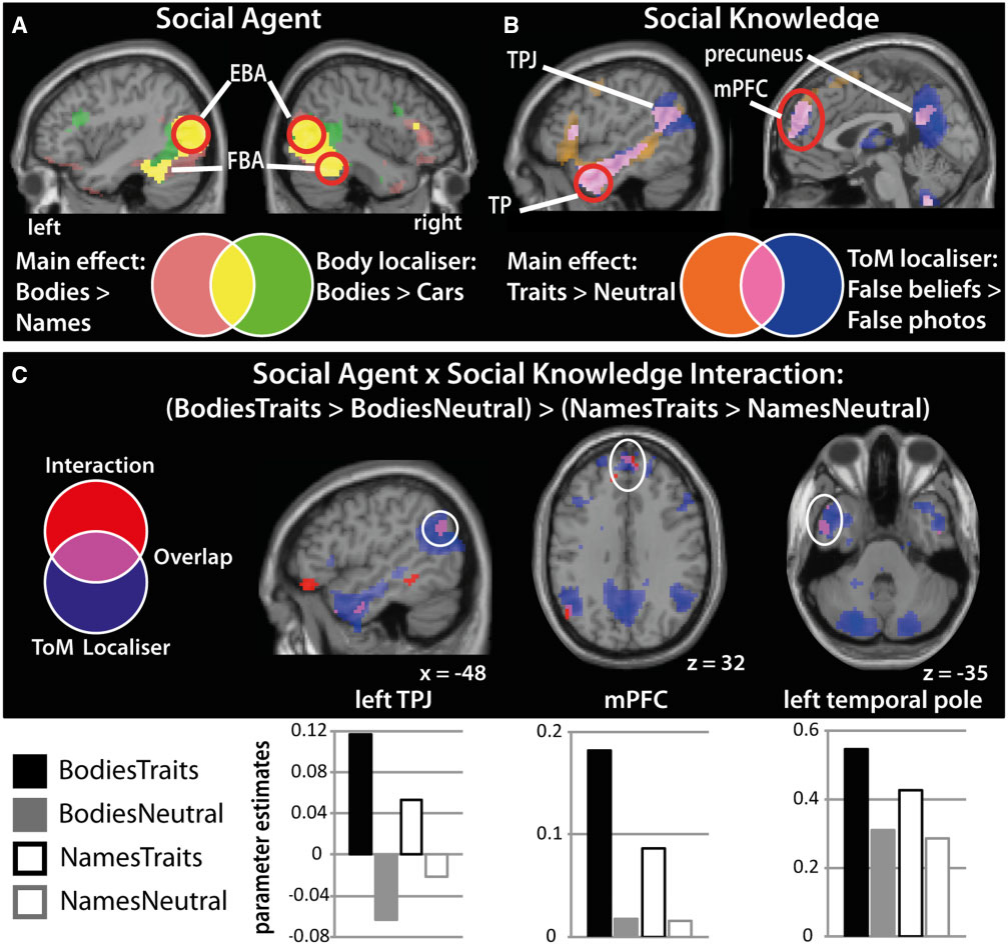
Results from the univariate analysis. **(A)** The main effect of Social Agent (Bodies > Names) revealed clusters of activity in bilateral OT cortices and bilateral FG. These clusters overlapped with the EBA and FBA as identified with the body-localiser (Bodies > Cars: green). Overlap is shown in yellow. **(B)** The main effect for Social Knowledge (Traits > Neutral) revealed clusters of activity in mPFC, bilateral TPs, precuneus and left TPJ. These clusters overlapped with the ToM-network as identified with the ToM-localiser (False Beliefs > False Photographs: blue). Overlap is shown in pink. **(C)** The Social Agent by Social Knowledge interaction ([BodiesTraits > BodiesNames] > [NamesTraits > NamesNeutral]) revealed a clusters in mPFC, left TP, and left TPJ, which overlapped with the ToM-localiser (overlap is shown in pink). Parameter estimates were extracted from a 4 mm sphere around the peak coordinate Abbreviations: EBA, extrastriate body area; FBA, fusiform body area; TPJ, temporoparietal junction; TP, temporal pole; mPFC, medial prefrontal junction.

There was a main effect of social agent (Bodies > Names; Figure 3A) in bilateral OT cortices [overlapping with Extrastriate Body Area (EBA) and surviving FWE cluster correction] and bilateral FG [overlapping with Fusiform Body Area (FBA) and surviving FWE cluster correction]. There was also a main effect of social knowledge (Traits > Neutral; Figure 3B) in mPFC, bilateral TPs, precuneus, and left TPJ, all of which overlapped with the ToM-localiser. The inverse contrasts for both main effects (Names > Bodies and Neutral > Trait) are reported in Supplementary Table S1.

##### Interaction

For the interaction between social agent and social knowledge [(BodiesTraits > BodiesNeutral) > (NamesTraits > NamesNeutral)] clusters emerged in left TPJ, mPFC, and left TP and all of these clusters overlapped with the ToM-localiser at the group-level (Table 1; Figure 3C). The parameter estimates illustrate a greater difference between trait and neutral statements when bodies rather than names are presented. More specifically, the effect of social knowledge (Traits > Neutral) is present for both social agents, but it is greater for bodies than names. These results demonstrate that brain regions defined by being engaged in reasoning about others’ mental states (social knowledge) emerge for the interaction term of the main task.

We also predicted that the person perception network would be engaged for the same interaction analysis, but we did not find this pattern of response at the initial threshold. To further explore this null result in EBA and FBA, we investigated the interaction term in body-selective regions at a more liberal threshold (*P* < 0.05, *k* = 10). Using this less conservative threshold, right FG showed the predicted interaction pattern and this cluster overlapped with the body-localiser (Supplementary Figure S1 and Table S2). In addition, there was a response in left middle temporal gyrus, but the location of this response was superior (*z* = 19) to the typical location of EBA or FBA. Due to the chance of the univariate response in right FG being a false positive, any interpretation is necessarily cautious. However, the main reason for performing the univariate interaction analysis was to identify seed regions that can be used subsequently to test our primary hypothesis using functional connectivity analyses. If the result in right FG is a false positive and it does not reflect the linking of body and trait information, then we should expect no functional coupling between right FG and the ToM-network in the functional connectivity analyses. The inverse interaction contrast [(NamesTraits > NamesNeutral) > (BodiesTraits > BodiesNeutral)] is reported in Supplementary Table S1.

### PPI analyses

Coordinates of overlap within individual participants were identified in left TPJ (*n* = 17), mPFC (*n* = 17), left TP (*n* = 15) and right FG (*n* = 19) (for more details, see Supplementary Table S3). Our prediction was that person perception and person knowledge networks would show coupling as a function of our task. To test this prediction, for each seed region separately, we used the same interaction term for our PPI analysis as was previously used in the univariate analysis [(BodiesTraits > BodiesNeutral) > (NamesTraits > NamesNeutral)].

**Fig. 4.**
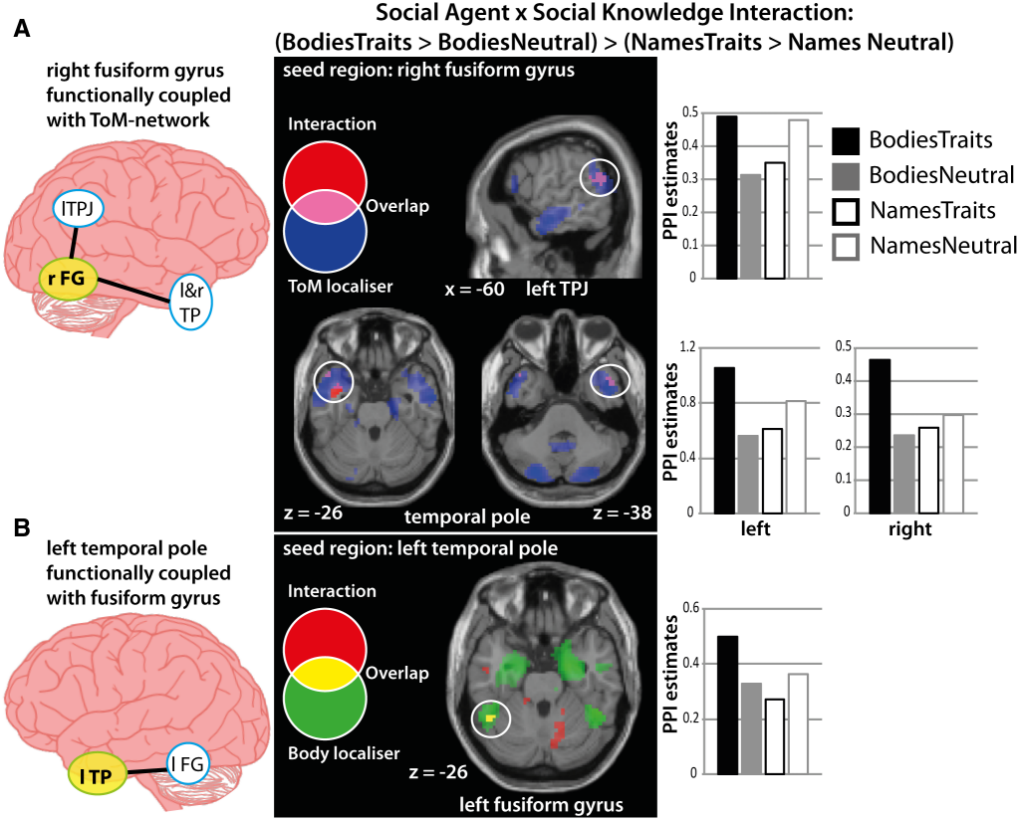
Results from the PPI analysis. Seed regions were identified based on clusters emerging from the social agent by social knowledge interaction at the univariate level (including right FG—see Supplementary Table S1). These regions were either part of the person perception network (right FG) or person knowledge network (mPFC, left TP and left TPJ) as defined by body and ToM localisers, respectively. In four separate PPI analyses, each identified region from the univariate analysis was used as a seed region with the social agent by social knowledge interaction term as the contrast of interest. Clusters emerging from these analyses reveal the strength of correlation over time between activity in that cluster and that in the seed region as a function of the task. These PPI parameter estimates are extracted from a 4-mm sphere around the peak coordinate. **(A)** PPI analyses revealed that seed region right FG (solid yellow circle) showed functional coupling with nodes within the person knowledge network. Clusters in left temporoparietal junction and bilateral TP showed greater functional coupling with right FG when inferring traits about bodies than names (shown in red). These areas overlapped with the ToM-localiser (shown in blue; overlap is shown in pink). **(B)** PPI analyses revealed that seed region left TP (solid yellow circle) showed functional coupling with left FG (shown in red). This area overlapped with the body-localiser (shown in green; overlap shown in yellow). Abbreviations: FG, fusiform gyrus; lTPJ, left temporoparietal junction; TP, temporal pole.

Both right FG and left TP showed the predicted pattern of functional coupling with person perception or knowledge networks (Table 2; Figure 4). Figure 4A shows that the response in left TPJ and bilateral TP has greater functional coupling with right FG when social knowledge (Trait > Neutral) is present for bodies, but not names. Additionally, these clusters all overlapped with the ToM-localiser. As such, there is overlap between the clusters that show coupling with right FG when inferring a trait about a body and when reasoning more generally about others’ mental states.

In addition, left TP showed greater functional coupling with a region of left FG when social knowledge (Trait > Neutral) is present for bodies, but not names (Figure 4B). Furthermore, this cluster in left FG overlapped with the body-localiser. As such, there is overlap between a cluster that shows coupling with left TP when inferring a trait about a body and when perceiving bodies in general.

The other seed regions, left TPJ and mPFC, did not show the predicted pattern of functional coupling with person perception networks. Therefore, the pattern of functional coupling observed between person perception and person knowledge networks when linking a trait to a body is not a general one that applies to every region within these two networks; instead, it is specifically tied to bilateral FG and parts of the ToM-network (left TPJ and bilateral TPs).

## Discussion

When being introduced to someone, one forms an impression based on what you’ve heard about her character (e.g. ‘She volunteers in a hospital’) as well as her physical appearance (e.g. tall and thin). Although much research has investigated the neural circuits involved in perceiving what another person looks like (person perception), as well as what one knows about that person (person knowledge), it is unclear how the human brain links these different pieces of information about a person’s identity together. We demonstrate that anatomically and functionally distinct brain circuits exchange signals during the formation of identity representation. Specifically, brain circuits that represent aspects of another person’s physical appearance, such as body shape and posture, are linked to brain circuits that engage when reasoning about another person’s trait-based character, such as whether they are friendly, helpful or generous. These data support the view that a ‘who’ system for social cognition spans perceptual and inferential mechanisms and that these mechanisms communicate to each other when forming a representation of another’s identity.

### Limitations and future directions

From our results we cannot infer whether the observed functional connectivity profile is tied to a particular person (i.e. person-specific) in addition to being tied to a particular form (i.e. body more than name). Given the trial-unique combinations of social agents and social knowledge, it is plausible that the results reflect person-specific representations. However, from our results alone, we cannot rule out the possibility that our results solely reflect a more generic category-level representation (i.e. body more than name). In addition, previous research has shown that mPFC is sensitive to person-specific information (Hassabis *et al*., 2014; Welborn and Lieberman, 2014). Future work, therefore, could adapt the methods developed here to directly test the degree to which person perception and knowledge networks interact at different levels of person-specificity.

One possible limitation to our interpretation relates to the familiarity of names that we used, which prior work has investigated (Sugiura *et al*. 2006). All names in this study were unfamiliar to participants and as such it could have been more difficult to assign social knowledge to names than bodies. However, this difference is unlikely to explain our results for two reasons. Our main findings involve an interaction between agent and knowledge. Therefore, a greater difficulty assigning knowledge to names in general would apply to both types of knowledge (trait-based and neutral), rather than being preferentially tied to trait-based judgments more than neutral judgements. In addition, by using functional localisers, it becomes more difficult for a difference in difficulty alone to explain why body-selective patches were linked to the person knowledge network, unless body-selective areas are also involved for difficult of processing *per se.*

We also acknowledge limits to our methodology and design, which future work can build upon. First, functional connectivity analyses provide no direct insight into the underlying neural pathway that controls functional coupling between brain areas. As such, using measures of structural connectivity, it would be valuable for future research to investigate the neural pathways that underlie functional relationships between person perception and person knowledge systems. Second, it is conceptually possible that trait information is linked to names through functional links between the ToM-network and a neural representation of names. For instance, there may be functional links between ToM areas and a brain area processing words, such as the Visual Word Form Area (VWFA; Szwed *et al*., 2011). However, we do not have the same grounds for hypothesising links between the ToM-network and a ‘name’ system, as we do for links with body patches. In contrast to EBA and FBA, which show category-selectivity for bodies, there is no evidence that the VWFA, or any other set of brain regions, shows the same category-selectivity for names (more than other words). In addition, we did not design the study to test for neural links between the ToM-network and a neural representation of names. To do so, we would have needed a relevant localiser in order to accurately locate the VWFA in each individual participant (Glezer and Riesenhuber, 2013). This study, therefore, was not designed to address neural links between the representation of names and traits. These caveats aside, the interaction contrast that tests for clusters showing a greater response for trait inferences (Traits > Neutral) when reading a name compared with observing a body, showed no engagement of ToM-network or any clusters with coordinates near VWFA (Supplementary Table S1C). As such, the limited evidence we do have from the current study regarding this issue is not consistent with neural links between the ToM-network and the VWFA, but much more work is needed to pursue this line of research directly.

### Implications for neural circuits subserving person perception and person knowledge

Coupling of functional responses between distinct brain circuits suggests that person perception and person knowledge networks are not completely encapsulated and resistant to influence from other brain systems. Downing and Peelen (2011) proposed that the primary function of EBA and FBA is to perform a visual analysis of bodies, but that these regions also exchange signals with other brain circuits. This study, as well as others (Ewbank *et al*., 2011; Quadflieg *et al*., 2011; Zimmermann *et al*., 2013), are beginning to provide empirical support for this view by demonstrating that interactions between neural systems that are part of a broader cognitive landscape may upregulate or downregulate the response in body-selective cortex.

### Linking person perception and person knowledge during social interactions

Neuroimaging research has identified patches of cortex selective for the perception of faces, bodies, and places as well as for thinking about other people’s thoughts (Downing *et al*., 2001; Spiridon *et al*., 2006; Kanwisher, 2010). Although these data have provided evidence for functional segregation within the human brain, it has not been clearly established how neural signals across multiple sites are integrated (Friston and Price, 2001; Friston *et al*., 2003). In the current experiment, we show that perceptual signals in the ventral visual stream are linked with inferential signals in the ToM-network. Specifically, we show that parts of the FG, which are involved in processing body shape and posture (Downing and Peelen, 2011), exchange signals with TPJ and TPs, which form part of a circuit that is involved in making inferences about others’ thoughts and traits (Frith and Frith, 1999; Saxe and Kanwisher, 2003; Mitchell, 2009; Van Overwalle, 2009). Moreover, we show that this exchange of signals is specifically tuned to situations when one is confronted with a combination of information that is relevant for both person perception and person knowledge networks (i.e. bodies, not names; traits, not neutral statements). As such, the pattern of functional connectivity is not generic to any form of social agent, such as someone we may read about in a novel; instead, it is tuned to an inference that is coupled to a body more than a name. On a broader level, these results provide empirical evidence to support the view that a ‘who’ system for social cognition, which establishes and maintains a global representation of another’s identity, comprises category-specific brain circuits that exchange signals (Haxby *et al*., 2000; Ishai 2008; Moeller *et al*. 2008; Ramsey *et al*., 2011; Collins and Olson, 2014).

## Funding

This work was funded by a grant from the Economic and Social Research Council (ES/K001884/1 to R.R.).

## Supplementary data

Supplementary data are available at *SCAN* online.

*Conflict of interest*. None declared.

Supplementary Data
