# Supplementary material for: Linking person perception and person knowledge in the human brain
Source: Soc Cogn Affect Neurosci. 2016 Feb 25;11(4):641–51. doi: 10.1093/scan/nsv148 (PMC4814794; doi:10.1093/scan/nsv148)
Supplement: Supplementary Data [file supp_11_4_641__index.html]

Linking person perception and person knowledge in the human brain — Supplementary Data 

# Linking person perception and person knowledge in the human brain

## Supplementary Data

files

- Supplementary Data - doc file
