# Supplementary material for: Linking person perception and person knowledge in the human brain
Source: Soc Cogn Affect Neurosci. 2016 Feb 25;11(4):641–51. doi: 10.1093/scan/nsv148 (PMC4814794; doi:10.1093/scan/nsv148)
Supplement: Supplementary Data [file supp_nsv148_Supplementary_Figure_S1.doc]

**Supplementary Figure S1.**

Results from the univariate analysis at a reduced threshold (p<.05, k=10). The Social Agent by Social Knowledge interaction ([BodiesTraits > BodiesNames] > [NamesTraits > NamesNeutral]) revealed a clusters right fusiform gyrus, which overlapped with the body-localiser (overlap is shown in yellow). These parameter estimates are extracted from a 4 mm sphere around the peak coordinate within the body-localiser.

**Supplementary Table 1.** Results from the univariate analysis: a) the main effect of social agent (Names > Bodies), b) the main effect of social knowledge (Neutral > Traits), and c) the social agent by social knowledge interaction [(NamesTraits > NamesNeutral) > (BodiesTraits > BodiesNeutral)].

| Region | Number of voxels | *T* | Montreal Neurological Institute coordinates | | |
| --- | --- | --- | --- | --- | --- |
|  |  |  | x | y | z |
| ***a) Main effect Social Agent: Names > Bodies*** | | | | | |
| **Left thalamus extending into hippocampus** | **325** | **6.42** | **-9** | **-34** | **13** |
|  |  | **5.41** | **39** | **-43** | **1** |
|  |  | **5.13** | **-18** | **-43** | **7** |
| **Right angular gyrus extending into right inferior parietal lobule** | **189** | **6.26** | **60** | **-55** | **37** |
|  |  | **5.79** | **57** | **-49** | **49** |
|  |  | **4.80** | **54** | **-58** | **46** |
| **Left supramarginal gyrus** | **508** | **6.18** | **-45** | **-43** | **37** |
|  |  | **4.89** | **-36** | **-55** | **46** |
|  |  | **4.72** | **-30** | **-61** | **40** |
| Right middle temporal gyrus | 130 | 5.79 | 63 | -19 | -14 |
|  |  | 3.68 | 60 | -4 | -11 |
| **Left insula** | **461** | **5.36** | **-45** | **11** | **1** |
|  |  | **4.89** | **-51** | **5** | **10** |
|  |  | **4.60** | **-42** | **5** | **19** |
| Right insula | 35 | 5.13 | 39 | 17 | 4 |
| **Left middle orbital gyrus** | **208** | **4.76** | **-33** | **47** | **4** |
|  |  | **4.14** | **-30** | **53** | **13** |
| Left middle frontal gyrus | 28 | 4.67 | -21 | 32 | 37 |
| Left supplementary motor area | 123 | 4.60 | -12 | -1 | 67 |
|  |  | 4.30 | -3 | 5 | 55 |
| Left precentral gyrus | 58 | 4.60 | -30 | -10 | 52 |
| Right middle frontal gyrus | 48 | 4.39 | 33 | 38 | 25 |
|  |  | 4.19 | 30 | 56 | 4 |
|  |  | 3.77 | 33 | 50 | 19 |
| Right caudate | 16 | 4.28 | 21 | 20 | 7 |
| Left middle temporal gyrus | 33 | 4.13 | -66 | -31 | -2 |
| Right cerebellum | 17 | 4.07 | 27 | -79 | -50 |
|  |  | 3.64 | 36 | -76 | -50 |
| Caudate | 54 | 3.90 | 3 | 5 | 16 |
|  |  | 3.59 | -6 | -1 | 22 |
| Left middle frontal gyrus | 17 | 3.21 | -42 | 29 | 31 |
|  |  | 3.15 | -36 | 26 | 25 |
| ***b) Main effect Social Knowledge: Neutral > Traits*** | | | | | |
| Left middle occipital gyrus | 52 | 5.91 | -36 | -85 | 31 |
| Left calcarine gyrus | 171 | 5.61 | -15 | -64 | 22 |
| Left fusiform gyrus | 169 | 5.60 | -33 | -37 | -20 |
| **Right calcarine gyrus** | **375** | **5.27** | **12** | **-61** | **19** |
|  |  | **4.50** | **-9** | **-70** | **49** |
|  |  | **3.87** | **9** | **-73** | **43** |
| Left middle frontal gyrus | 64 | 4.53 | -21 | 17 | 49 |
| Left inferior frontal gyrus (pars triangularis) | 120 | 4.48 | -36 | 32 | 16 |
|  |  | 4.37 | -27 | 35 | -17 |
| **Right middle frontal gyrus** | **369** | **4.36** | **33** | **32** | **34** |
|  |  | **4.19** | **30** | **35** | **43** |
|  |  | **4.14** | **42** | **44** | **16** |
| Right middle cingulate cortex | 116 | 4.26 | 3 | -28 | 40 |
|  |  | 3.53 | -6 | -28 | 34 |
| Left orbitofrontal cortex | 15 | 3.85 | -27 | 59 | -8 |
| Right anterior cingulate cortex | 10 | 3.83 | 15 | 23 | 16 |
| Right medial frontal gyrus | 38 | 3.77 | 9 | 35 | 31 |
|  |  | 3.16 | 15 | 35 | 19 |
| Left insula | 35 | 3.57 | -39 | -10 | -11 |
| Left intraparietal sulcus | 16 | 3.45 | -57 | -43 | 49 |
| Right hippocampus | 12 | 3.27 | 24 | -37 | -8 |
|  |  | 2.88 | 33 | -37 | -17 |
| Right postcentral gyrus | 19 | 3.17 | 63 | -40 | 43 |
| Left postcentral gyrus | 12 | 3.12 | -60 | -31 | 34 |
| ***c) Interaction: Social agent * knowledge [(NamesTraits > NamesNeutral) > (BodiesTraits > BodiesNeutral)]*** | | | | | |
| Left superior temporal gyrus | 56 | 4.65 | -42 | -16 | 4 |
|  |  | 3.24 | -51 | -13 | -5 |
| Right caudate | 12 | 4.47 | 24 | 5 | 25 |
| Left postcentral gyrus | 111 | 4.44 | -63 | -25 | 22 |
|  |  | 3.83 | -48 | -28 | 13 |
|  |  | 3.68 | -45 | -25 | 34 |
| Right cuneus | 154 | 4.15 | 12 | -73 | 43 |
|  |  | 4.08 | 15 | -70 | 31 |
|  |  | 3.85 | 12 | -76 | 25 |
| Right superior parietal lobule | 48 | 4.06 | 15 | -31 | 43 |
| Left middle occipital gyrus | 25 | 4.02 | -24 | -100 | -8 |
| Right inferior parietal lobule | 40 | 3.85 | 60 | -37 | 34 |
| Right inferior parietal lobule | 75 | 3.82 | 39 | -31 | 40 |
|  |  | 3.27 | 42 | -46 | 43 |
|  |  | 2.90 | 33 | -46 | 40 |
| Left cerebellum | 16 | 3.53 | -27 | -37 | -41 |
|  |  | 3.11 | -33 | -40 | -47 |
| Right middle frontal gyrus | 10 | 3.19 | 42 | 56 | 13 |

Note: Regions surviving a voxel-level threshold of p<.005 and 10 voxels are reported. Areas in bold survive FWE cluster correction for multiple comparisons. Subclusters at least 8 mm from the main peak are listed.

**Supplementary Table 2.** Exploratory analysis of the univariate interaction analysis in body-selective regions. The social agent by social knowledge interaction [(BodiesTraits > BodiesNeutral) > (NamesTraits > NamesNeutral)] is masked by the body-localiser.

| Region | Number of voxels | *T* | Montreal Neurological Institute coordinates | | |
| --- | --- | --- | --- | --- | --- |
|  |  |  | x | y | z |
| ***Interaction: Social agent * knowledge*** | | | | | |
| Right fusiform gyrus | 44 | 2.94 | 45 | -46 | -11 |
|  |  | 2.84 | 48 | -43 | -8 |
|  |  | 2.28 | 48 | -43 | 1 |
| Left middle temporal gyrus | 12 | 2.60 | -45 | -64 | 19 |

Note: Regions surviving a voxel-level threshold of p<.05 and 10 voxels are reported. Subclusters at least 8 mm from the main peak are listed. These results are exclusively masked by the NamesNeutral > NamesTraits contrast to make sure that any interaction result does not include (Neutral > Traits) when paired with names.

**Supplementary Table 3**. Details of individual subjects’ overlap between social agent and social knowledge interaction and the ToM (for seed regions left temporoparietal junction, medial prefrontal cortex, and left temporal pole) and body (for right fusiform gyrus) localisers. The details in this table are not intended to be interpreted on their own.

| Seed-region | *Interaction threshold at which overlap was found in individual subjects* | | | | | | | | |
| --- | --- | --- | --- | --- | --- | --- | --- | --- | --- |
|  | p<.001 | p<.005 | p<.01 | p<.05 | p<.1 | p<.2 | p<.3 | p<.4 | p<.5 |
| Left temporoparietal junction (n=17) | 1 | 1 | 2 | 10 | - | 1 | - | 2 | - |
| Medial prefrontal cortex (n=17) | 1 | 1 | - | 15 | - | - | - | - | - |
| Left temporal pole (n=15) | - | 2 | 4 | 8 | - | - | 1 | - | - |
| Right fusiform gyrus (n=19) | - | 7 | - | 2 | 2 | 3 | 5 | - | - |
